# Extraction and Elevation of Cell-Free DNA under Mastitis and Heat Stress in Dairy Cattle

**DOI:** 10.3390/ani13091487

**Published:** 2023-04-27

**Authors:** Yumei Chen, Zaheer Abbas, Lirong Hu, Ling Kang, Xiao Tan, Qing Xu, Yachun Wang

**Affiliations:** 1 Institute of Life Science and Bioengineering, Beijing Jiaotong University, Haidian, Beijing 100044, China; 19121601@bjtu.edu.cn (Y.C.); zaheerabbas@bjtu.edu.cn (Z.A.); lingkang3187@163.com (L.K.);; 2 School of Animal Science and Technology, China Agricultural University, Haidian, Beijing 100193, China; b20193040324@cau.edu.cn

**Keywords:** cell-free DNA, extraction method, mastitis, heat stress, Holstein dairy cows

## Abstract

**Simple Summary:**

This is the first study investigating the effects of mastitis and heat stress on plasma cfDNA levels in dairy cattle. We established a complete system for the extraction of cell-free DNA from the plasma of dairy cows with the high-yielding method of the TIANamp Micro DNA Kit. The concentration was determined by real-time quantitative PCR, and the results showed that cfDNA is related to mastitis and heat stress in dairy cattle.

**Abstract:**

In this study, four methods (phenol-chloroform protocol, sodium iodide kit, QIAamp DNA Blood Mini Kit, and TIANamp Micro DNA Kit) were used to extract cell-free DNA (cfDNA) from cattle blood, and the yield and purity of cfDNA varied in four different methods from 0.36 to 0.84 ng/mL for yield and 0.67 to 1.80 (A260/A280) for purity. Compared with other methods, the TIANamp Micro DNA kit performed better in both cfDNA amount and purity (*p <* 0.05); furthermore, blood cfDNA levels were significantly increased in Holstein dairy cows under the influence of heat stress (*p* < 0.01) and mastitis (*p* < 0.0001), which showed a potential power to discriminate mastitis (AUC = 0.99, 95% CI = 0.97 to 1.00) or heat stress (AUC = 0.86, 95% CI = 0.73 to 0.98) in cows. In brief, we established a complete experimental system for the extraction of cfDNA from cattle blood based on the high-yielding method of the TIANamp Micro DNA Kit and showed the effect of mastitis and heat stress on cfDNA levels in cattle blood for the first time. Our findings suggested that cfDNA in cattle blood may be a useful marker to measure mastitis and heat stress in dairy cattle.

## 1. Introduction

Cell-free DNA (cfDNA) is an extracellular free nucleic acid existing in body fluids [1]. Compared to common nucleic acids, cfDNA are shorter, fewer in number, and more easily degraded [2]. Numerous characteristics of cfDNA revealed cell death due to apoptosis, necrosis, or a disease condition as its primary origin. Various studies have established that cfDNA can act as a useful biomarker in the diagnosis of cancers, prenatal testing, and organ transplantation status monitoring [3,4,5]. In particular, it has the advantage of asymptomatic early detection. Usually, the cfDNA level in patient plasma is higher than that in a healthy individual; moreover, the cfDNA concentration changes with the level of trauma, injury, and exercise [6,7]. Previous studies also claimed a strong correlation of cfDNA with diseases or physiological responses in humans. In contrast to the widespread use of cfDNA as a biomarker in human prenatal and cancer diagnosis, only a small number of studies have employed cfDNA in early pregnancy diagnosis in dairy cows, and the potential application of cfDNA in bovine illnesses and stress response has yet to be discovered.

Bovine mastitis and heat stress have seriously impaired the production and health of dairy cows, causing subsequent limitations in the sustainable development of the dairy industry [8,9]. Bovine mastitis, an inflammatory reaction to intramammary infection is one of the most prevalent and hazardous diseases in dairy cows [8]. Literature reported that cfDNA is associated with inflammatory responses such as systemic lupus erythematosus and pancreatitis [10]. We hypothesize that mastitis as a form of inflammation may cause changes in blood cfDNA in cattle. Considering a thermo-humidity index (THI) value greater than 72, cattle experience heat stress, resulting in a cascade of reactions that further reduce productivity, reproductive performance, and immunity [9,11]. The molecular effect of heat stress in cows is challenging due to the complexity of animal metabolic and physiological responses. Since heat stress causes significant damage to cells or tissues [12], cfDNA is released and subsequently enters the blood, increasing the cfDNA level.

The low concentration and small fragment size of cfDNA limit its usefulness as a source of clinical biomarkers in blood, thus, better purification methods are needed; however, the extracting methods of cfDNA used to assess its plasma/serum concentration are diverse, and no universal protocol standardizations are established [13]. Further, most of the methods have been tested and adjusted only for human samples, not for cattle. Therefore, this study is carried out to successfully extract cfDNA, and to evaluate if mastitis or heat stress produces changes in blood cfDNA in cattle.

## 2. Materials and Methods

### 2.1. Animals’ Selection and Sampling

The use of animals and experiments were in agreement with the Animal Experimentation Ethics Committee at Beijing Jiaotong University, Beijing, China (Code ID: SS-QX-2014-06). A total of 3 animal groups were selected: set 1: 6 healthy Holstein cows for the cfDNA extraction methods; set 2: 18 healthy and 18 clinical mastitis Holstein cows for the relationship between mastitis and cfDNA level; set 3: 18 healthy in summer as the heat stress group (July, THI > 78) and 18 in autumn as the thermo-neutral group (November, 45.5 < THI < 68). The THI was measured using an automatic weather station (Model: P4581, Comet System S.R.O., Bezrucova, Czech Republic) located within the lactating cows’ pen. These animals were raised at the Sanyuan dairy farm in Beijing, China. The cows were kept under basic managerial practices, fed three times a day in a scattered hurdle with a full mixed ration, and had full access to fresh drinking water. The experimental cows were housed in a semi-open barn with sprinklers above the feeding line and fans above the stall. In summer, cooling sessions were held during feeding times. Free-stall fans and sprinklers were operational during the hottest period in the area. Cows were allowed to rest in the barn-free yard outside at night, depending on the weather. The animals with clinical mastitis were diagnosed by a veterinarian, and the samples were directly obtained from the farm. According to the diagnosis history, the cows showed clear signs of swelling and inflammation of the udder, fever, reduced feed intake, and the animal avoided lying to reduce pressure on the udder.

**Figure 1:**
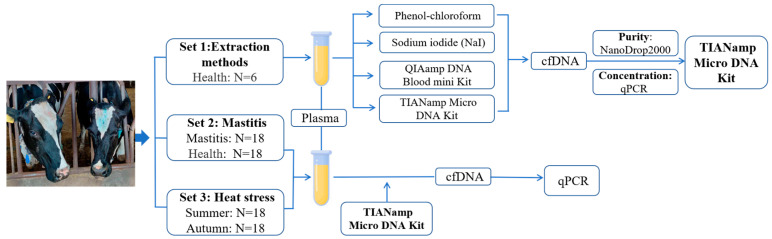
The analytical workflow performed in this study.

The venous blood of each animal was collected with uncoagulated EDTA and centrifuged at 1600 g (4 °C) for 10 min to separate the plasma. Aliquots were prepared within 3 h after collection and centrifugation and stored at −80 °C until further use. Figure 1 shows the detailed description of animal sets and experiment flow used in the current study.

### 2.2. Extraction of cfDNA

Plasma samples were thawed at 4 °C and then centrifuged at 4000× *g* for 15 min to further remove blood cells. CfDNA was extracted from the supernatant soon after the separation and rinsed with TE buffer (Qiagen, Hilden, Germany) with a volume of 50 μL. Extraction of the cfDNA from the aliquots of plasma was carried out with four different procedures including TIANamp Micro DNA Kit (TIANGEN Biotech, Beijing, China), QIAamp DNA Blood Mini Kit (Qiagen, Hilden, Germany), sodium iodide (NaI, Wako Pure Chemical Industries Ltd., Osaka, Japan) protocols and phenol-chloroform. All methods were used according to the manufacturer’s instructions.

### 2.3. Quantification of cfDNA

The purity of cfDNA was assessed with NanoDrop2000, and the concentration of cfDNA was measured with quantitative real-time PCR (BIO-RAD CFX-96, Bio-Rad Laboratories, Inc., Feldkirchen, Germany) (NYSE: BIO and BIOb). The forward primer sequences of 18SrRNA were: 5′-GTAACCCGTTGAACCCCATT-3′ and the reverse were 5′-CGCTACTACCGATTGGATGG-3′. Absolute equivalent amounts of DNA in each sample were determined by a standard curve with five ten-fold serial dilutions of genomic DNA from the peripheral blood of a healthy cow. Serial dilutions of an external standard and water blanks were included in every run. The qPCR was carried out on an Applied Biosystem^®^ Step One Plus™ (Applied Biosystems, Foster City, CA, USA), using iTaq™ Universal SYBER Green Supermix (Bio-Rad laboratories GmbH, Feldkirchen, Germany). Amplification was performed in a 20 µL reaction volume with 2 µL of cDNA (10 ng/µL), 10 µL of 1 × SYBER Green Master Mix (Bio-Rad laboratories GmbH, Feldkirchen, Germany), 0.6 µL of forwarding primers, 0.6 µL of reverse primers, and 6.8 µL of RNase-Free H_2_O. All qPCR experiments were carried out in triplicate.

### 2.4. Statistical Analysis

Quantitation of cfDNA was calculated based on mean ± standard deviation (SD). Differences were evaluated by Student’s *T*-test. Significance was established at *p* < 0.05. Receiver operating characteristic (ROC) analysis was performed using IBM SPSS Statistics 22.0.

## 3. Results

### 3.1. Evaluation of Extraction Methods for cfDNA from Cattle Blood

Four methods were used to extract cfDNA from cattle blood, as shown in Table 1. Each method captured cfDNA from the cattle blood despite variations in the cfDNA yield and quality, which revealed blood cfDNA in cattle is a common presence, similar to humans. Next, the amount of cfDNA extracted from cattle blood descended in the order of TIANamp Micro DNA Kit (0.84 ng/mL), QIAamp DNA Blood Mini Kit (0.79 ng/mL), sodium iodide (NaI) protocols (0.49 ng/mL) and phenol-chloroform (0.36 ng/mL). The TIANamp yielded the highest concentration of cfDNA compared to the three other methodologies (*p <* 0.05) (Table 1). In terms of cfDNA purity, the TIANamp Micro DNA Kit showed the best performance (*p <* 0.05) (A260/A280, 1.80 ± 0.25) as shown in Table 1. Given the high cfDNA extraction efficiency in cattle blood, the TIANamp Micro DNA kit was considered optimal and used in the following study.

### 3.2. Effect of Mastitis and Heat Stress on the cfDNA Level in Cattle Blood

**Figure 2:**
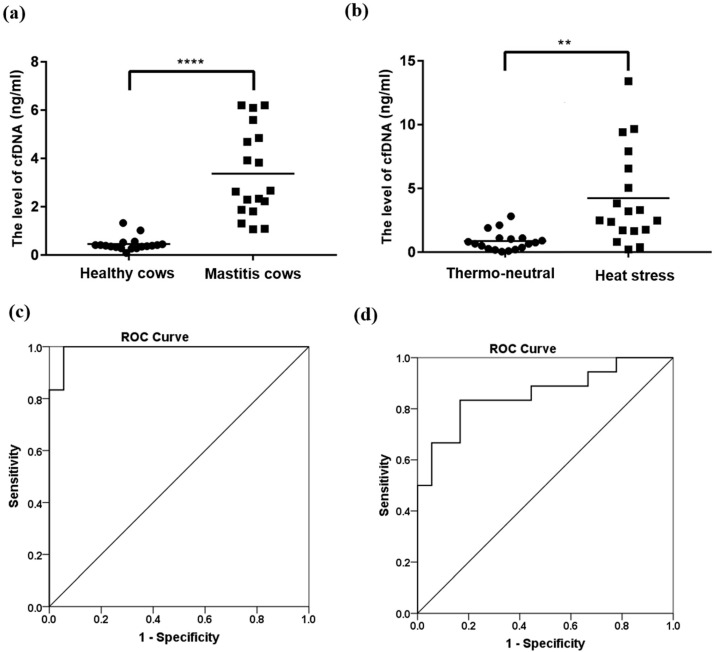
The effect of mastitis and heat stress on the cfDNA levels in cows. (**a**,**b**): Levels of cfDNA determined by 18SrRNA genes; (**a**): healthy and mastitis cows (**** *p* < 0.0001); (**b**): cows in heat stress and thermo-neutral (** *p* < 0.01). (**c**,**d**): ROC curve of cfDNA; (**c**): healthy and mastitis cows (AUC = 0.99); (**d**): cows in heat stress and thermo-neutral (AUC = 0.86).

To study the possible effect of mastitis and heat stress on the cfDNA level in cattle blood, cfDNA concentrations in all used samples were determined with quantitative real-time PCR by taking the 18SrRNA gene as a determination object. The average concentration of cfDNAs was 3.37 ng/mL in the 18 mastitis cows and 0.46 ng/mL in the 18 healthy cows. The levels of cfDNA in mastitis cows were significantly higher (*p* < 0.0001) than that in healthy cows (Figure 2a). Similarly, there was a statistically significant difference (*p* < 0.01) in cfDNA concentration between heat stress (4.23 ng/mL) and thermo-neutral animals (0.86 ng/mL), so heat stress could increase the cfDNA content of dairy cows (Figure 2b). Further, the receiver operating characteristic curve (ROC) analysis was performed to test the accuracy of cfDNA levels in estimating mastitis and heat stress. The accuracy of cfDNA levels both in mastitis and heat stress was excellent, indicated by the area under the curve (AUC) being 0.99 (*p* < 0.001, 95% CI = 0.97 to 1.00; Figure 2c) and 0.86 (95% CI = 0.73 to 0.98; Figure 2d) in mastitis and heat stress, respectively. The results of the quantitative and ROC analysis support that cfDNA levels can be used as a potential indicator for the detection of mastitis and heat stress in dairy cows.

## 4. Discussion

Cell-free DNA in blood represents a valuable biological marker in human clinical research and diagnosis [4]. Almost every study on cell-free DNA concludes that tissue necrosis or apoptosis is the primary source of its origin. DNA deterioration is considered a defining feature of apoptosis, in which chromosomal DNA is first sliced into large fragments and then into multiple small fragments [1,14,15]. Compared with the genomic DNA in whole blood, the content of cfDNA in plasma is lower, and the fragments are shorter, which makes it easy to lose during its quantification process, thus optimizing the extraction efficiency can help cfDNA-based assays perform better [16,17]. Considering the previous limited information about cfDNA in cattle blood, in this study, we used four different methods to isolate cfDNA from cattle blood under different conditions. The TIANamp Micro DNA Kit showed convincing results compared to others in extraction efficiency and purity; moreover, the operation of this kit was simple and safe. TIANamp is a micro-column adsorption kit, and the silicone membrane in the micro-column selectively combines with DNA resulting in a significant increase in the yield and purity of DNA. By using the TIANamp kit in the current study, we not only obtained cfDNA from the cattle blood but also evaluated the application of cfDNA in cattle under heat stress and mastitis.

CfDNA levels in plasma are increased in a variety of pathological and environmental conditions, causing systemic inflammation, tissue damage, necrosis, or apoptosis [18]; however, plasma cfDNA concentration is exceptionally high in some conditions and coincides with the prognosis [3,19]. With the wide application of cfDNA as a biomarker in human prenatal and cancer diagnoses, the research on cfDNA in animals is increasing. A recent study in dogs reported an increased level of cfDNA in neoplastic dogs compared to the ones without neoplasia, and among all the neoplastic cases, the concentration of cfDNA in the lymphoid neoplasia group was significantly higher [20]. In cattle, it was found that there were some different sequence tags in the serum between pregnant and non-pregnant dairy cows at different stages, and the distribution of cfDNA was significantly different [21]. Research about the diagnosis of bovine pregnancy showed that cfDNA could be used as a marker to detect early pregnancy in cattle [22]. Gordon et al. found there were sequences related to disease in the cfDNA of elk and cattle infected with transmissible spongiform encephalopathies, which may be helpful for early diagnosis of TSE disease in cattle and elk [23]. Temporal pattern changes of cfDNA was explored in a newborn piglet model of hypoxia, and the concentration of cfDNA in the cerebrospinal fluid of the piglet exposed to hypoxia was higher than that of the control group after intervention [24]. Filev et al. showed that the plasma cfDNA concentration increased in rats subjected to stress [25]. Considering the above studies, a strong correlation between cfDNA and various animal diseases or stress responses is evident in animals.

The udder inflammation or heat stress causes severe tissue damage, leading to cell death and apoptosis [26,27,28]. Mastitis is an inflammatory condition of the mammary gland parenchyma due to various forms of infections and environmental factors [8,26]. Animals with mastitis respond through complicated mechanisms, including various molecular and cellular defenses to preserve biological equilibrium and to endure quick and significant changes caused by infection [29]. Such balance is interrupted by the excessive release of reactive oxygen species (ROS), which is harmful to the molecular structure of cell membranes, resulting in functional and molecular changes in afflicted cells and, as a result, tissue and organ dysfunction [30]. Furthermore, heat stress can cause mitochondrial damage and oxidative stress, which causes severe structural and functional damage to the cells and initiates apoptosis and necrosis-related pathways [31]. The PI3K/Akt signaling pathway mediates physiological functions such as cell proliferation, autophagy, and apoptosis, and it has been reported that this pathway is disrupted during heat stress [28]. Consequently, as a result, cfDNA may be released into the extracellular environment leading to a higher level of cfDNA than normal in the blood. Furthermore, the statistical analysis showed good discrimination power in mastitis or heat stress in cows according to the ROC analysis; however, the difference in AUC between mastitis (0.99) and heat stress (0.86) in cows indicated that the specificity and sensitivity of cfDNA for the diagnosis of different traits may be different. Overall, our data indicated cfDNA may be used as a potential marker for the detection of mastitis or heat stress without visual symptoms in dairy cattle.

Similar to other studies, this study showed that in the future, plasma cfDNA can be used in early screening, evaluation of therapeutic response, and prognosis in a variety of diseases in dairy cattle. Moreover, increased cfDNA levels could be attributable to the severity of illness or poor treatment outcomes in mastitis or heat stress cows. Because of certain differences between individuals and only a small sample of participants, it is suggested that more samples should be used to further verify the results of this experiment in the future. Considering the shortcomings of this study we are aiming to further work on large sample sizes and to find out whether the quantification of cfDNA could be monitored for therapeutic responses and prognosis of mastitis or heat stress and follow-up.

## 5. Conclusions

We evaluated and established a complete experimental system for extraction of cfDNA from cattle blood based on the high-yielding method using the TIANamp Micro DNA Kit and further investigated the effects of mastitis and heat stress on cfDNA levels in cattle blood for the first time. Our findings suggested that cfDNA in cattle blood may be a useful marker to measure mastitis and heat stress in dairy cattle.

## Figures and Tables

**Table 1:** Yield and purity of cfDNA in plasma of cows.

Method	Quantitative Real-Time PCR	NanoDrop2000
	cfDNA Yield (Mean ± SD, ng/mL)	cfDNA Purity (A260/280)
Phenol-chloroform	0.36 ± 0.16 ^a^	0.67 ± 0.15 ^a^
Sodium iodide (NaI)	0.49 ± 0.13 ^a^	1.01 ± 0.06 ^a^
QIAamp DNA Blood Mini Kit	0.79 ± 0.14 ^b^	1.31 ± 0.17 ^b^
TIANamp Micro DNA Kit	0.84 ± 0.16 ^b^	1.80 ± 0.25 ^c^

Note: ^a,b,c^ Different letters indicate significant differences between the methods (*p* < 0.05).

## Data Availability

Not applicable.
